# New Population and Life Expectancy Estimates for the Indigenous Population of Australia's Northern Territory, 1966–2011

**DOI:** 10.1371/journal.pone.0097576

**Published:** 2014-05-27

**Authors:** Tom Wilson

**Affiliations:** Queensland Centre for Population Research, School of Geography, Planning and Environmental Management, The University of Queensland, Brisbane, Queensland, Australia; Iran University of Medical Sciences, Iran (Islamic Republic Of)

## Abstract

**Background:**

The Indigenous population of Australia suffers considerable disadvantage across a wide range of socio-economic indicators, and is therefore the focus of many policy initiatives attempting to ‘close the gap’ between Indigenous and non-Indigenous Australians. Unfortunately, past population estimates have proved unreliable as denominators for these indicators. The aim of the paper is to contribute more robust estimates for the Northern Territory Indigenous population for the period 1966–2011, and hence estimate one of the most important of socio-economic indicators, life expectancy at birth.

**Method:**

A consistent time series of population estimates from 1966 to 2011, based off the more reliable 2011 official population estimates, was created by a mix of reverse and forward cohort survival. Adjustments were made to ensure sensible sex ratios and consistency with recent birth registrations. Standard life table methods were employed to estimate life expectancy. Drawing on an approach from probabilistic forecasting, confidence intervals surrounding population numbers and life expectancies were estimated.

**Results:**

The Northern Territory Indigenous population in 1966 numbered between 23,800 and 26,100, compared to between 66,100 and 73,200 in 2011. In 1966–71 Indigenous life expectancy at birth lay between 49.1 and 56.9 years for males and between 49.7 and 57.9 years for females, whilst by 2006–11 it had increased to between 60.5 and 66.2 years for males and between 65.4 and 70.8 for females. Over the last 40 years the gap with all-Australian life expectancy has not narrowed, fluctuating at about 17 years for both males and females. Whilst considerable progress has been made in closing the gap in under-five mortality, at most other ages the mortality rate differential has increased.

**Conclusions:**

A huge public health challenge remains. Efforts need to be redoubled to reduce the large gap in life expectancy between Indigenous and non-Indigenous Australians.

## Introduction

Considerable policy attention in Australia is focused on addressing the many socio-economic dimensions of disadvantage experienced by its Aboriginal and Torres Strait Islander, or Indigenous, peoples. A voluminous literature exists on disadvantage in terms of health, mortality, education, income, labour force, housing, the justice system, and other areas [1], [2]. In response, the federal and state and territory governments have jointly agreed a set of targets to “close the gap” between Indigenous and non-Indigenous Australians across a range of indicators [3], including “closing the life expectancy gap within a generation” and “halving the gap in mortality rates for Indigenous children under five within a decade” [4]. Unfortunately, however, Indigenous disadvantage is also firmly entrenched in the realm of statistics, with many Indigenous-focused demographic and other socio-economic measures subject to uncertainty, questioning, and regular revisions which are frequently inconsistent with previous figures [5]. Understanding the precise extent of disadvantage and its causes, formulating policy solutions and monitoring progress is difficult without a foundation of trustworthy statistics, and in particular, population estimates which form the denominators of so many indicators. This paper argues that improving the trustworthiness of statistics is not just about increasing accuracy, however, but also about providing an indication of the extent of uncertainty.

Census counts and population estimates of the Indigenous population have long suffered from coverage and quality limitations. Up to and including the 1966 Census, Indigenous people were only enumerated in order to exclude them from official population totals. The first attempt at a full enumeration occurred in the 1971 Census. Coverage is thought to have gradually improved with successive censuses, but even in 2011 the Post-Enumeration Survey estimated a net undercount of 17% for the Indigenous population nationally [6]. Variable census coverage has produced an inconsistent population time series, with sudden discontinuities in growth between successive five-year intercensal periods [7]. Similarly, population change in individual cohorts often appears implausible [8]. Research on the extent of changes to Indigenous reporting in the census over time has been very limited due to the lack of data permitting its direct measurement. However, in December 2013 ABS released the Australian Census Longitudinal Dataset (ACLD) which comprises a 5% sample of 2006 Census records linked with the 2011 Census [9]. The extent of changes to Indigenous status reporting can now be examined, and relevant data are presented in this paper.

Since the early 1990s the Australian Bureau of Statistics has also published Indigenous Estimated Resident Populations (ERPs) [10], [11], [12], [13]. These official figures are attempts at estimating the ‘true’ usually resident Indigenous population taking into account census underenumeration and a variety of other factors. Until recently ABS labelled them ‘experimental’ to emphasise the lower quality and greater uncertainty relative to the main set of ERPs for the Australian population as a whole. Although probably closer to the ‘real’ Indigenous population than census counts, Indigenous ERPs also tend to suffer the same sort of temporal inconsistencies as census data. Nonetheless, the 2011 ERPs are probably the best estimates of Australia's Indigenous population to date, due to a much expanded Post-Enumeration Survey (PES) in remote Indigenous communities compared to earlier censuses, and an improved method of linking census and PES records to estimate undercount [14].

Unfortunately Indigenous births and deaths data in Australia are of lower quality than ERPs, and suffer from significant undercounting. ABS advises that whilst most Indigenous deaths are likely to be recorded, some of them will not be correctly identified as Indigenous [15]. To investigate the extent of mortality underreporting, ABS recently undertook a Census Data Enhancement Indigenous Mortality Study which linked census and death records over a 13 month period following the 2011 Census [16]. It concluded that, at a national level, 82% of Indigenous deaths over the study period were identified as Indigenous in death records.

However, the Northern Territory is the one jurisdiction in Australia widely acknowledged to possess high quality Indigenous births and deaths data. Indigenous affairs – and therefore statistics – are far more prominent because about 30% of its population identifies as Indigenous compared to no more than 5% in any other state and territory [17]. The Northern Territory Government has invested considerable resources in its statistical systems with the intention of obtaining complete coverage of Indigenous people. The recent ABS Mortality Study estimated coverage of Indigenous deaths in the mortality statistics in the Northern Territory to be complete (in fact, the estimate was just over 100%!) [16]. High quality deaths data in the Northern Territory extend all the way back to 1967 thanks to a painstaking categorisation of individual death records undertaken by Condon et al. [18]. Indigenous births data are more restricted in extent and are available only from 1988.

Given this data environment what is the best approach to creating a consistent set of Indigenous population estimates for the Northern Territory over the last few decades? Use of census counts or Indigenous ERPs based on separate censuses would be unwise because of variations in coverage and population accounting inconsistencies. The absence of reliable births data prior to the late 1980s prohibits the use of inverse projection methods [19]. Given the reliability of deaths data and the 2011 Indigenous ERPs, the logical approach is to ‘start’ in 2011 and work backwards in time by taking into account deaths and migration numbers. In fact, this reverse survival approach was used by Condon et al. [18] in their preparation of 1966–2001 estimates with the assumption of zero net migration at all ages.

This paper updates and extends that work. It produces a set of mid-year age-sex population estimates for the Indigenous population of the Northern Territory for years ending in 1 and 6 (census years) over the period 1966–2011. It modifies the 2011 ERPs to obtain a more plausible population in the 0–4 age group and more reasonable sex ratios in some adult ages. It also includes non-zero migration as part of the reverse survival calculations. Uncertainty in the 2011 ERPs and in the deaths and migration numbers is explicitly considered so that the time series of estimates is expressed in the form of confidence intervals. The paper additionally contributes a new dataset of one of the most important social indicators, life expectancy at birth, for each intercensal period (covering 1st July in one census year to 30th June at the next census year 5 years later). This is also presented with confidence intervals.

Following this introduction the paper discusses the data sources used, including their strengths and weaknesses, and various definitions of the Indigenous population embedded in these data. It then describes the methods applied to adjust the 2011 ‘starting’ populations, produce estimates back to 1966, and estimate life expectancy at birth. This section also describes how estimates of uncertainty were applied to the population and life expectancy figures. The resulting population estimates and life expectancy at birth numbers form the focus of the next section. Concluding remarks include some comments on the policy implications of this research.

## Data

The study required 2011 Estimated Resident Populations (ERPs), and deaths and net migration counts for 1966v2011 in order to construct the new set of population estimates. Births data were also acquired for the period 1991–2011 in order to make adjustments to the 0–4 year old ERP. Data on changes to Indigenous status reporting in the Northern Territory between the 2006 and 2011 Censuses were extracted from the Australian Census Longitudinal Dataset (ACLD).

### 2011 population estimates

The preliminary set of population estimates obtained were the 30th June 2011 Indigenous ERPs by sex and five year age group [14]. These estimates are based on 2011 Census usually resident counts with adjustments for census net underenumeration, non-response to the Indigenous status question, the time difference between the census (9th August) and mid-year, and people temporarily overseas on census night.

What is the quality of these 2011 Indigenous ERPs? In the absence of a robust independent population benchmark it is difficult to give a definite answer. They are *probably* the best official Indigenous population estimates produced to date given a larger post-enumeration survey sample in Indigenous communities than in earlier censuses and, for the first time, the application of automated data linking census and Post-Enumeration Survey returns which reduced the proportion of unlinked records [6]. Nonetheless, the 2011 Indigenous ERPs contain some inconsistencies both internally and with the demographic components of change. In the Northern Territory they contain obvious age and sex imperfections for which adjustments were also needed (discussed below).

**Figure 1:**
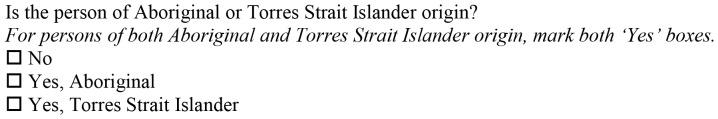
The standard ABS question on Indigenous status.

The definition of Indigenous in these estimates is the ABS working definition embedded in the census question shown in Figure 1. Individuals who tick either or both of the ‘Yes’ boxes are categorised by the ABS as Indigenous. Thus individuals' Indigenous status is based on self-reporting or the reporting of the individual filling out the census form on behalf of others, such as parents for young children. It is therefore dependent on the interpretation of the question by census respondents, and answers may vary over time and from place to place. Fortunately the recently released ACLD provides some information on this phenomenon. Note that unlike some other countries, such as the UK, there is no mixed ethnicity category, nor can individuals be both Indigenous and non-Indigenous, unlike New Zealand for example where the census allows the reporting of multiple ethnicities.

### Changes to Indigenous status reporting

Data on the extent to which individuals changed their reported Indigenous status between the 2006 and 2011 censuses were obtained from the ACLD. A cross-tabulation of Indigenous status from the two enumerations for the Northern Territory is presented in Table 1. The data reveal the extent of change to Indigenous status reporting in the Northern Territory to be small, with an even smaller net change. Note that the table displays unweighted counts from the ACLD, and clearly the sample is too small to be disaggregated by age and sex. Caution needs to be exercised in interpreting these figures not only because of the sample size, but also due to the presence in the ACLD of some false links and unmatched records. Nonetheless, it seems likely that net Indigenous status reporting change is small enough in the Northern Territory to be assumed to be zero for the purposes of creating historical estimates. The only exception concerns very young children, as explained below.

**Table 1:** Changes to reported Indigenous status between 2006 and 2011, Northern Territory.

	Indigenous status in 2011		
Indigenous status in 2006	Indigenous	Non-Indigenous	Not stated
Indigenous	1,420	47	12
Non-Indigenous	33	4,604	38
Not stated	8	59	3

Note: Data shown are unweighted counts from the Australian Census Longitudinal Database 5% sample.

Source: [41].

### 0–4 year olds

**Figure 2:**
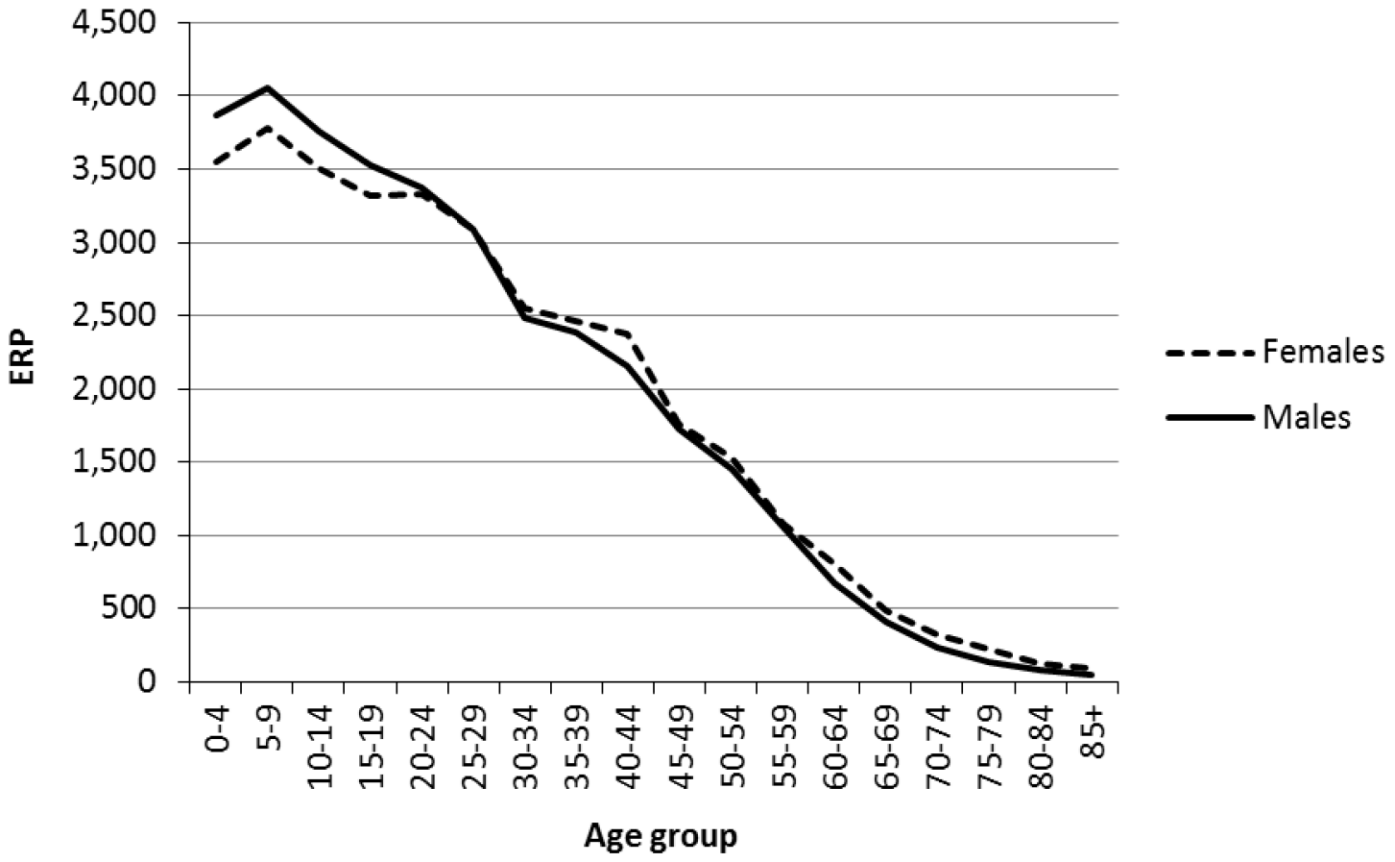
Indigenous Estimated Resident Populations for the Northern Territory, 2011.

Figure 2 presents official ABS ERPs by five year age group and sex for the Northern Territory Indigenous population at 30th June 2011. As can be seen in the graph, 0–4 year olds are less numerous than 5–9 year olds. This is a little puzzling because the numbers of Indigenous birth registrations over the intercensal periods 2001–2006 and 2006–2011 are very similar. Whilst deaths and net migration numbers experienced by these two cohorts differ slightly, they are not nearly enough to account for the relative sizes of the 0–4 and 5–9 age groups in 2011.

**Figure 3:**
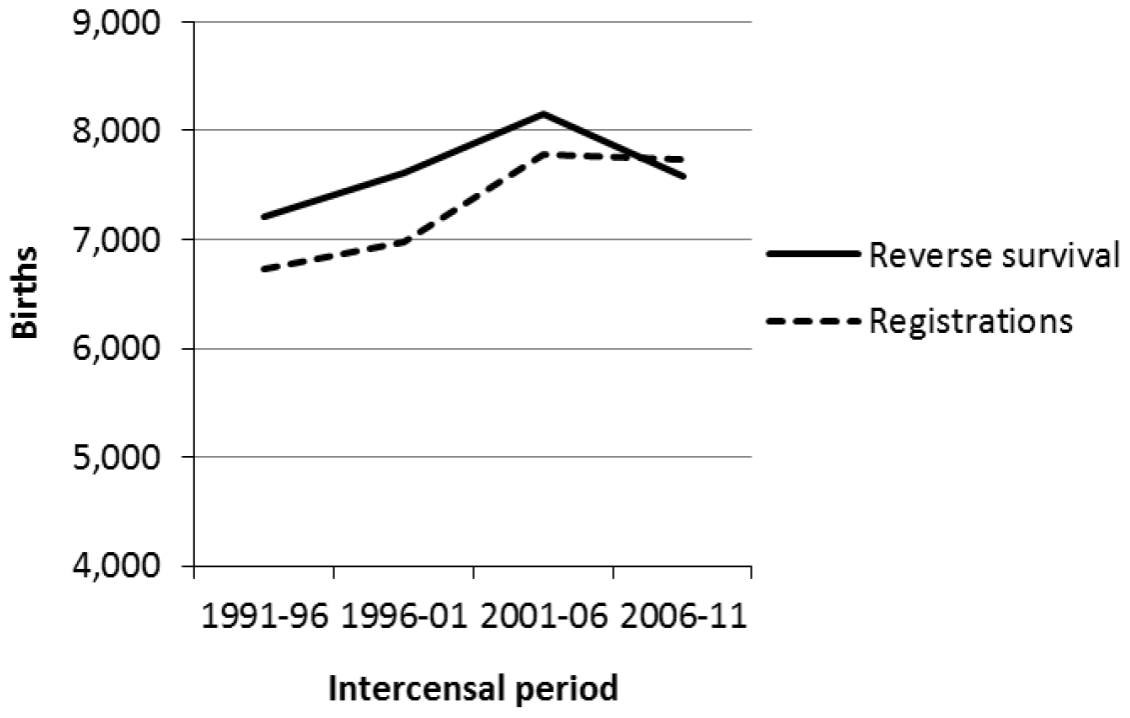
Northern Territory Indigenous births, 1991–1996 to 2006–2011.

A comparison of registered Indigenous births with those estimated by reverse population accounting over the last four intercensal periods helps shed some light on this problem (Figure 3). Applying reverse survival to the 0–4 ERP in 2011 – taking into account 2006–11 cohort deaths and net migration – results in fewer births than were actually registered for 2006–2011. Yet similar reverse survival starting with 2011 ERPs in age groups 5–9, 10–14 and 15–19 generates ‘reverse estimated’ births for 2001–06, 1996–2001 and 1991–1996 respectively which are 5–9% higher than the registrations. Repeating the exercise by reverse surviving ABS's 2006 and 2001 ERPs (not shown) replicates the pattern in Figure 3.

Is this an undercount of births and/or an overcount in the ERP? Or is there some change in reported Indigenous status occurring amongst infants? Writing about the Australian Indigenous population as a whole, Gray and Gaminiratne [20] were the first to suspect it was the latter, arguing “a large proportion of young children that are not enumerated as Aboriginal come to be classified as Aboriginal at slightly older ages” (p. 3). They suggested it was due to mixed Indigenous/non-Indigenous couples being unsure of how to answer the question on Indigenous status in the census for their newly-born infants. Gray and Gaminiratne argued that by the time of later censuses, the child's identity was clearer and was more likely to be reported as Indigenous.

More data are now available to test this theory. The results of a recent ABS report on ‘Understanding the increase in Aboriginal and Torres Strait Islander counts, 2006–2011’ support Gray and Gaminiratne's idea [21]. Nationally, most of the increase in Indigenous census counts amongst the cohort aged 0–4 in 2006 and 5–9 in 2011 occurred to mixed Indigenous/non-Indigenous couples or where both parents were non-Indigenous or where the Indigenous status of one parent was not recorded. The cohort of infants with two Indigenous parents experienced only a slight increase between 2006 and 2011.

**Figure 4:**
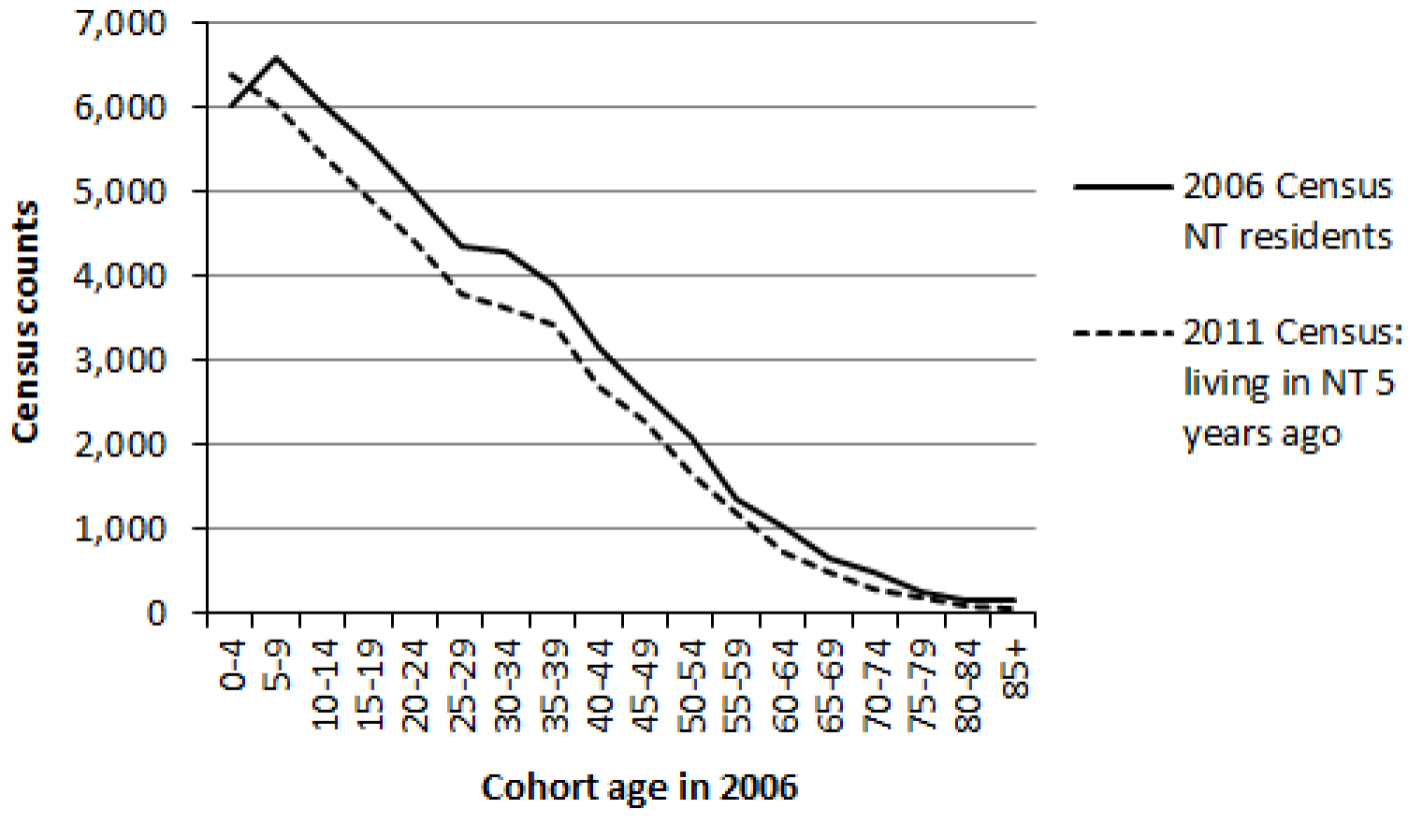
Comparison of Northern Territory (NT) Indigenous census counts in 2006 with 2011 Census counts of Indigenous persons living in the Northern Territory 5 years earlier.

As noted above, the sample size from the ACLD is too small to measure the extent of this phenomenon for specific age groups in the Northern Territory. However, indirect supporting evidence is displayed in Figure 4. It shows 2006 Census counts of Indigenous persons resident the Northern Territory alongside 2011 Census counts of those who stated that they were living in the Northern Territory five years earlier (i.e. at the time of the 2006 Census). The 2011 Census figures are derived from the question on place of residence five years ago. They are lower primarily because of non-response to the question on usual address five years ago and depletions due to mortality – but with the notable exception of the cohort aged 0–4 in 2006. The 2006 Census count of this cohort *should* be higher than the 2011 count. The pattern is consistent with a change of reported Indigenous identity occurring for cohorts between the ages of 0–4 and 5–9. The Methods section describes how this issue was handled in the creation of consistent historical population estimates.

### Deaths

Prior to 1988 Indigenous status was not collected on Northern Territory death notification forms. However Condon et al. [18] examined individual death records for 1967–1988 and inferred Indigenous status from other information on the forms, such as a distinctive Indigenous name, a place of birth in a remote Indigenous community, and marital status recorded as ‘tribally married’. A validation of this approach using 1991 death records in which Indigenous status was temporarily hidden revealed it be accurate, with the possibility of very slight under-identification of Indigenous deaths. Condon et al. [18] were therefore able to assemble a robust dataset of Northern Territory Indigenous deaths covering the period 1967 to 2001. Aggregate deaths data by sex and age groups 0, 1–4, 5–9, …, 80–84 and 85+ for each year 1967 to 2001 were supplied for this study by Dr John Condon of the Menzies School of Health Research. Deaths for the second half of 1966 were assumed to be half the number which occurred in 1967. More recent Indigenous deaths data for the intercensal periods 2001–2006 and 2006–2011 were obtained from ABS in the same abridged life table age groups as above.

The coverage of Indigenous death registration is regarded as high. As mentioned earlier, the ABS Census Data Enhancement Indigenous Mortality Study for 2011–2012 estimated full coverage of Indigenous deaths in the Northern Territory. This situation has, fortunately, existed for some time. In their study using Indigenous data for the late 1980s and early 1990s, Luther et al. [22] viewed the Northern Territory Indigenous deaths coverage as “virtually complete”. In fact Condon et al. [18] report that “death registration in the Northern Territory is believed by registration authorities to be relatively complete from the late 1950s”.

The definition of Indigenous in the deaths data is based on the inferral method of Condon et al. [18] for deaths up to 1988 and death registration information thereafter. ABS [15] cautions that the way in which the question on the deceased's Indigenous status is recorded depends on who completes the paperwork, the perceived use of the information, as well as personal and cultural factors. Although the ABS Mortality Study found a very high level of agreement in deaths and census data regarding Indigenous status, there are likely to be minor definitional differences between census and ERP data on the one hand and deaths on the other.

### Births

Like deaths, Indigenous status has only been collected on Northern Territory birth notification forms since 1988. Since this time coverage of the Indigenous population is believed to be virtually complete [18]. The definition of Indigenous in the data is dependent on the ABS practice of defining “a birth as being an Aboriginal and Torres Strait Islander birth where at least one parent reported themselves as being an Aboriginal person, Torres Strait Islander, or both on the birth registration form” [23]. Although census evidence suggests that the vast majority of babies born to mixed Indigenous/non-Indigenous couples are regarded by the parents as Indigenous, this is not universally the case [8]. The ABS definition is unfortunate because, first, it is imposed by statisticians and not the individuals to which it refers, and second, it introduces a theoretical inconsistency with population and deaths data [24]. In practice the inconsistency appears to be small for the case of the Northern Territory.

### Migration

Successive censuses have revealed immigration by the Northern Territory's Indigenous population to be tiny, and it is reasonable to assume emigration flows to be of similar size. Net international migration can therefore be regarded as negligible. Interstate migration, however, can not. Five year interval census interstate migration flows by sex and five year age group were obtained for the intercensal periods 1966–1971 to 2006–1011 from the ABS. These data are derived from census questions on current place of usual residence and place of usual residence five years earlier.

More uncertainty surrounds the quality of interstate migration data than the other demographic components. More traditionally-oriented Indigenous people may conceptualise usual address and geographical mobility differently from mainstream western society [25]. It is not fully understood to what extent the high levels of circular and temporary mobility undertaken by many Indigenous people [26] are inadvertently captured in the census (permanent) migration data. In addition, census net interstate migration statistics are used in this paper as if they were event data, even though, strictly, they are fixed-interval transition data [27]. For *net* migration, the differences should not be too large.

## Methods

This section describes how estimates of the Northern Territory's Indigenous population by sex and five year age group over the period 1966 to 2011 were created by a mix of reverse and forward cohort survival. Life tables were also created for five year intercensal periods. It then explains how estimates of uncertainty were produced for both population numbers and life expectancy at birth. Data outputs have been deposited in the Australian Data Archive under ADA identifier number 01261 with a catalogue entry at http://ada.edu.au/ada/01261.

### Reverse cohort survival

**Figure 5:**
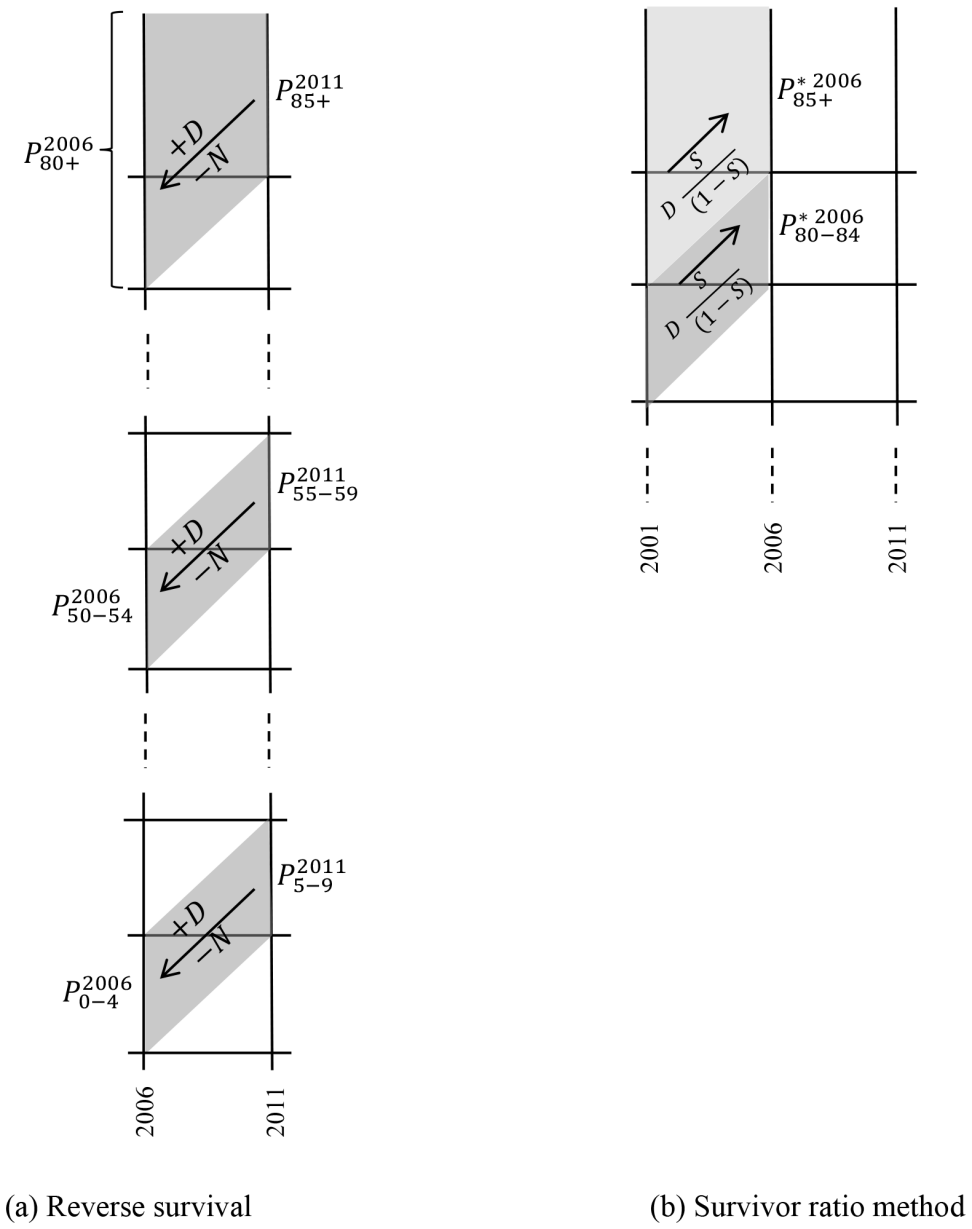
Lexis diagram illustration of (a) reverse cohort survival estimation and (b) the survivor ratio method for calculating preliminary 80–84 and 85+ populations.

Population estimates for all five year age groups up to 75–79 and 80+ were created using a reverse cohort population accounting equation with known death and net migration numbers. For example, the population aged 50–54 at 30th June 2006 was calculated as:
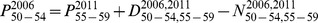
where P denotes population, D cohort deaths, N cohort net interstate migration, “2006,2011” the time period between 1st July 2006 and 30th June 2011, and “50–64, 55–59” the cohort aged 50–54 in 2006 and 55–59 in 2011. Figure 5(a) illustrates the method in Lexis diagram format. Net interstate migration data are strictly five-year fixed interval transition net migration but they are used here as an approximation of movement-type migration. International migration of the Northern Territory Indigenous population has been ignored and, given the limited changes to reported Indigenous status shown in Table 1, changes to reported Indigenous identity were assumed to be zero at ages 5 and above.

Deaths data were available only up to the cohort aged 80+ to 85+ for most of the period of study, thus limiting use of the reverse accounting equation to age 80+. Had deaths been available for every cohort up to the highest age lived then reverse population accounting could have been used to estimate all age groups. Instead, the survivor ratio method was employed to complete the elderly population numbers by disaggregating the 80+ age group into 80–84 and 85+. A survivor ratio is the ratio of a cohort's population size at a particular time to its size (often five) years earlier. Usually the method is applied to calculate an up-to-date population of an old and nearly extinct cohort [28], [29]. In such cases a preliminary estimate of a nearly extinct cohort's population at age a and time t can be found with death counts and survivor ratios as:
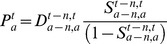
(1)where S denotes the survivor ratio. Because the survivor ratio for the period t-n,t is unknown it is assumed to be the same as that of the adjoining older cohort which has been calculated using the extinct cohort method [30]. To allow for improved survivorship over time, preliminary population estimates are constrained to an independent total deemed to be reliable, such as the official 90+ population, as recommended by Thatcher et al. [31]. For the purposes of this paper, however, the survivor ratio method was applied by borrowing ratios from adjoining *younger* cohorts.

Calculation of the 80–84 and 85+ populations via the survivor ratio method proceeded in three steps, and is described here with example equations for the 2006 estimates. First, survivor ratios for 2006–2011 were calculated:
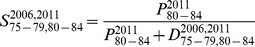
(2)and



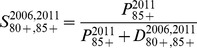
(3)Second, preliminary (*) populations aged 80–84 and 80+ in 2006 were estimated on the basis that the 2006–11 survivor ratios provided a good approximation of the 2001–2006 ratios:
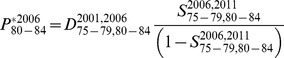
(4)and



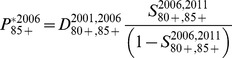
(5)Net migration at these high ages is zero and was excluded from the calculations. The Lexis diagram in Figure 5(b) illustrates the age-time location of these calculations. Third, the preliminary 80–84 and 85+ populations were constrained to sum to the 80+ estimate from the reverse population accounting. For 1966 a minor modification was necessary due to the lack of deaths for the preceding five years. Instead, deaths for 1966–1971 were used.

### Adjustments to the 0–4 year old ERPs

To adjust for the inconsistency between births and numbers of 0–4 year olds on the one hand and 5–9 year olds on the other a decision was made to inflate births for 2006–11 to match the Indigenous population likely to exist by the time the cohort reaches ages 5–9. The inflation factors of 1.065 for females and 1.086 for males were the average ratios of births estimated by reverse survival from 5–9, 10–14 and 15–19 age groups to official birth registration figures for 1991–96 to 2001–06. The key assumption here is one of stability in the ratio of ‘reverse survived’ to registered births. The new 0–4 year old population was then estimated by forward cohort survival of the ‘inflated’ births by subtracting deaths and adding net migration. The new 0–4 year old population was thus estimated as 8,157, an increase of 744, or 10%, over the ERP of 7,413. Consequently the total 2011 Indigenous population for the Northern Territory was revised upwards from 68,850 to 69,594.

However, it is important to point out that although this adjustment provides consistency in Indigenous definition between 2011 population estimates and reverse survival estimates for young children, it is not a perfect solution. It probably introduces a minor degree of inconsistency with deaths and net migration data. Although the extent of the inconsistency is probably small, without linking vital statistics with census records it is impossible to be certain.

### Adjustments to selected adult age group ERPs

Adjustments were also made to ERPs in selected age groups to obtain more plausible sex ratios. The 2011 sex ratios in ERPs for age groups 20–34 and 40–44 appeared too low, and once reverse cohort survival had been applied to estimate the number of cohort births it gave sex ratios at birth close to, or below, unity. The 40–44 age group was the most suspicious with a sex ratio in 2011 of 0.90 (surrounded on either side by age groups with ratios of 0.97 and 0.98) and a sex ratio at birth in 1966–71 of 0.99.

Sex ratios for the problematic cohorts were adjusted by accepting the initial number of reverse survived births of both sexes, imposing a normal sex ratio of birth of 1.06 to create revised numbers of male and female births, and then applying a forward accounting equation. The resulting sex ratios for these cohorts in 2011 and earlier years were more realistic. As a consequence the total Indigenous sex ratio was increased from 1.00 to 1.02.

### Life expectancy

Abridged life tables for intercensal periods 1966–1971 to 2006–2011 were calculated using the formulae set out in Preston et al. [32]. Comparative abridged life tables for the Australian population as a whole were also prepared for exactly the same time periods.

### Accounting for uncertainty

The above methods were applied to generate a set of Indigenous population estimates by age and sex, and life tables, spanning a 45 year period to 2011. Although the population and deaths data were believed to be of generally high quality, and the census net interstate migration of reasonable quality, the ‘true’ values of these demographic events may have been higher or lower than their recorded or estimated values. In order to communicate the uncertainty of population and life expectancy estimates to users, confidence intervals were constructed. For life expectancy at birth these confidence intervals cover both random variation (mentioned above) and data uncertainty.

Confidence intervals were generated in a manner similar to that of probabilistic population forecasting [33]. Starting with the adjusted 2011 populations, 5,000 reverse cohort survival simulations with randomly varying deaths and net migration were produced back to 1966. The 2011 starting populations were also selected at random from a distribution based on the ABS Post-Enumeration Survey standard error for the Northern Territory Indigenous population of 1,849 [6]. Recorded deaths and net migration numbers were both varied by multiplying by a term consisting of a factor 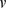 randomly varying around 1.0 in a normal distribution. For example, deaths for the cohort aged a at time t and a+5 at t+5 were modelled as:
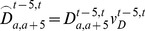
(6)where 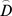 represents the randomly varied number of deaths and D the recorded number. For the first period factor v was calculated as
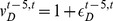
(7)and then assumed to follow a random walk backwards in time:
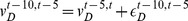
(8)with errors 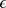 following a normal distribution with a mean of zero. For deaths, errors were assumed to have a standard deviation of 0.02, whilst the greater uncertainty surrounding net migration was reflected in a standard deviation set at 0.10. Whilst it would have been preferable to have available more evidence on which to base these assumptions, unfortunately this was not the case. So in the absence of such evidence these standard deviations were based on an overall judgement from the limited information available on quality discussed in the Data section. The resulting confidence intervals can therefore only be interpreted as indicative and approximate. Nonetheless, they do signal to users that Indigenous demographic statistics such as these are uncertain and should not be interpreted as overly precise. The sensitivity of alternative standard deviations for error is reported in Table S1. Confidence intervals for total population were generated by sorting all 5,000 simulations and selecting values at chosen fractiles along the distribution, such as at 0.975 and 0.025 for the upper and lower 95% bounds.

For life expectancy at birth 95% confidence intervals were created which cover both data uncertainty and random variation. The data uncertainty component was generated by estimating life expectancy at birth for all 5,000 simulations with varying deaths, migration and population. The random variation component was then estimated using the method developed by the UK Office for National Statistics [34], itself an adaptation of Chiang's [35] method of creating life expectancy confidence intervals. It should be noted that the confidence intervals cannot cover all sources of uncertainty in life expectancy, and should be regarded as indicative only.

## Results

### Populations

**Figure 6:**
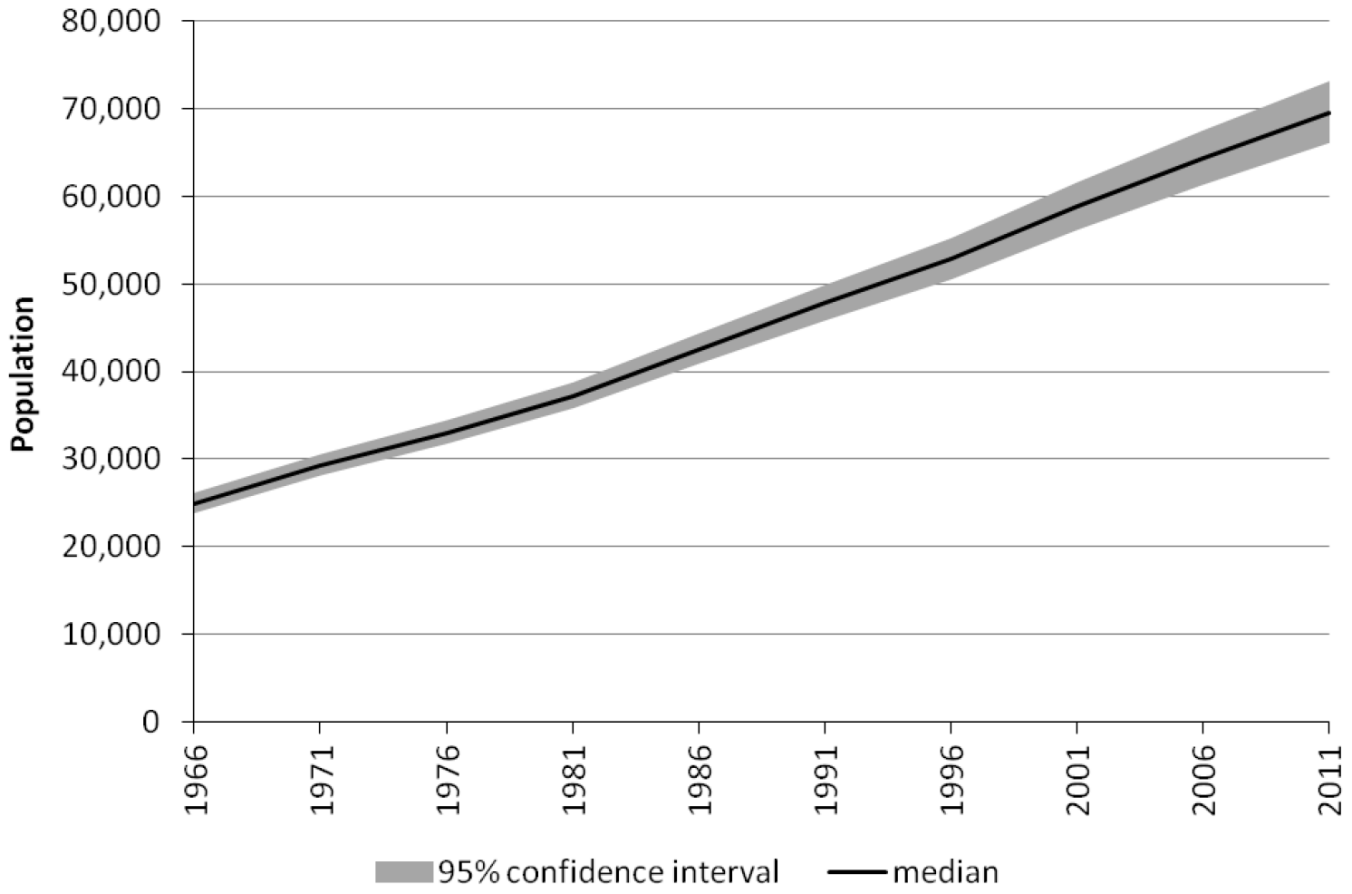
Estimates of the Northern Territory Indigenous population, 1966–2011.

Total estimates of the Northern Territory Indigenous population, along with 95% confidence intervals, are shown in Figure 6. They indicate growth from between 23,800 and 26,100 in 1966 to between 66,100 and 73,200 in 2011 (95% intervals). The median of the distribution rises from 25,000 to 69,600 over the period. These numbers translate to a growth rate of around 3% per annum at the beginning of the period, falling in an irregular trend to around half that by 2006–2011.

Unlike probabilistic population forecasts in which uncertainty increases with time from the launch year, the uncertainty of these estimates diminishes the further back from the 2011 population estimates one goes. This occurs partly because the randomly varying paths of deaths and net migration fluctuate to a fairly limited extent around the recorded numbers of deaths and net migrants and do not drift off on radically different trajectories. It is also due to the variation being proportional to the number of recorded deaths or net migrants, so that the smaller numbers of these demographic events in the earlier part of the study period result in smaller intervals.

How do the new set of population estimates compare with those published elsewhere? Table 2 provides the answers in terms of total population. Not surprisingly, given the similarity of methods, they are close to the 2001-based reverse survival estimates of Condon et al. [18]. The new estimates differ primarily due to the 0–4 year old adjustment in 2011 and net interstate migration. Census counts provide the lowest set of numbers and are between 12% and 28% lower than the new estimates. ABS ERPs based on the census counts, however, are in closer agreement, especially in 1996 and 2006 (and of course, 2011). What is surprising is the near alignment of the new estimates with those of the Northern Territory administration [36], at least over the limited part of the study period for which they are available. Both 1966 and 1971 administration totals fall within the 95% confidence interval of the new estimates.

**Table 2:** Comparison of the new Northern Territory Indigenous population estimates and growth rates with those published elsewhere.

	1966	1971	1976	1981	1986	1991	1996	2001	2006	2011
*30^th^ June populations*										
Author's estimates	24,960	29,300	33,063	37,242	42,598	47,821	52,838	58,851	64,400	69,593
Census count	21,119	23,253	23,748	29,081	34,733	39,888	46,362	50,785	53,662	56,777
ERP	n/a	n/a	n/a	n/a	n/a	43,273	51,876	56,875	64,005	68,850
Gray and Smith's minimal total Aboriginal populations	21,386	24,187	26,829	29,086	n/a	n/a	n/a	n/a	n/a	n/a
Condon et al. estimates	25,345	29,090	33,218	37,289	41,890	46,642	51,922	56,875	n/a	n/a
Luther et al.'s consistent correction estimates	n/a	n/a	n/a	n/a	37,175	41,234	n/a	n/a	n/a	n/a
NT administration estimates	24,120	28,500	n/a	n/a	n/a	n/a	n/a	n/a	n/a	n/a
*Annual average growth rates (%) over preceding 5 years*										
Author's estimates	n/a	3.2	2.4	2.4	2.7	2.3	2.0	2.2	1.8	1.6
Census count	n/a	1.9	0.4	4.1	3.6	2.8	3.0	1.9	1.1	1.1
ERP	n/a	n/a	n/a	n/a	n/a	n/a	3.6	1.8	2.4	1.5
Gray and Smith's minimal total Aboriginal populations	n/a	2.5	2.1	1.6	n/a	n/a	n/a	n/a	n/a	n/a
Condon et al. estimates	n/a	2.8	2.7	2.3	2.3	2.1	2.1	1.8	n/a	n/a
Luther et al.'s consistent correction estimates	n/a	n/a	n/a	n/a	n/a	2.1	n/a	n/a	n/a	n/a
NT administration estimates	n/a	3.3	n/a	n/a	n/a	n/a	n/a	n/a	n/a	n/a

Sources: [17], [18], [22], [36], [42], [43], [44], [45], [46], [47], [48], [49], [50], [51].

Annual average growth rates calculated from the various sets of population statistics are shown in the lower panel of Table 2. They demonstrate the hazards of estimating intercensal growth from population numbers based on separate censuses rather than an internally consistent time series. For example, over the 2001–2006 period annual average growth was 1.8% according to the new estimates, but other data series put growth at 1.1% and 2.4%.

**Figure 7:**
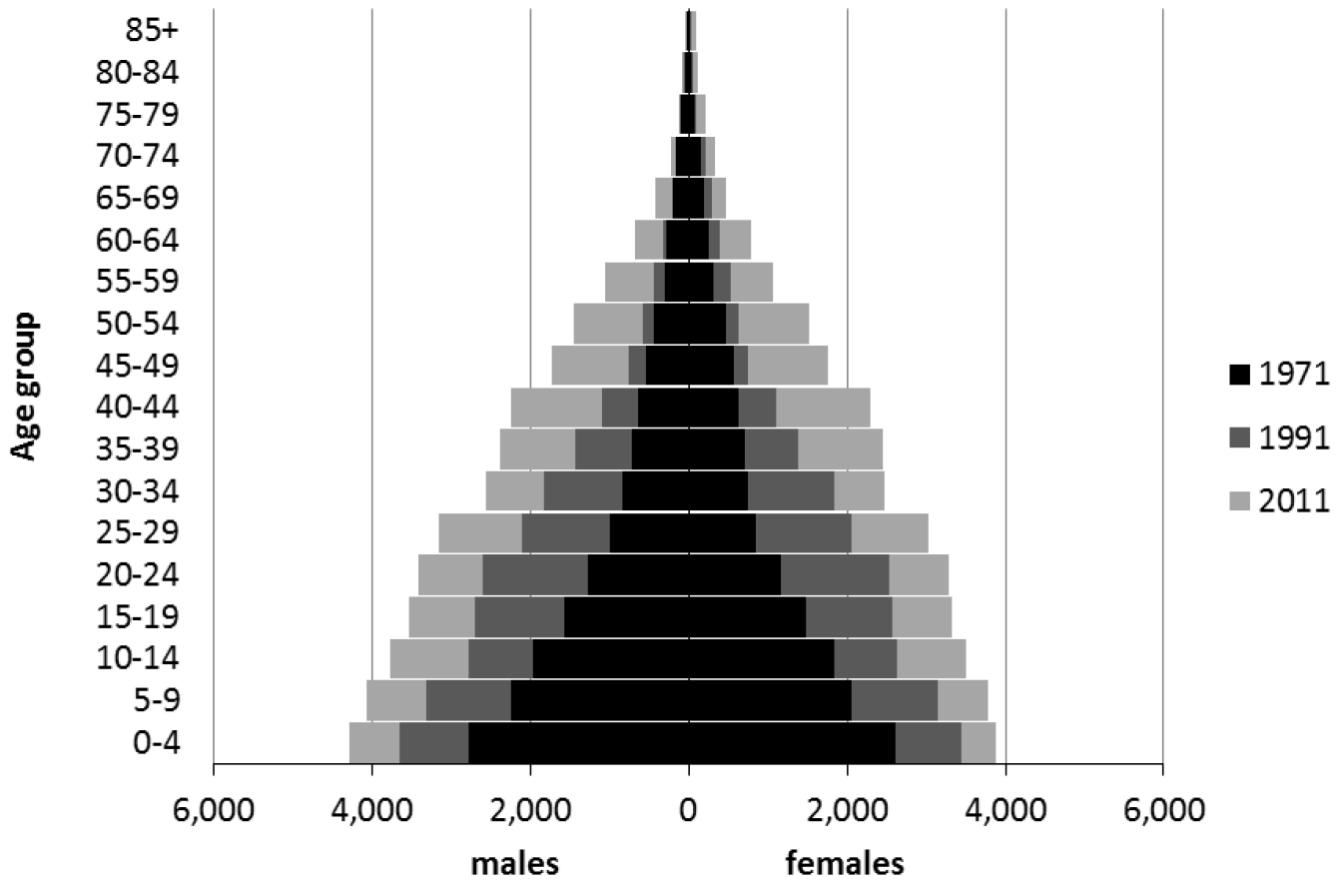
The age-sex structure of the Northern Territory Indigenous population, 1971, 1991 and 2011.

Age-sex population estimates for selected years from the median of the estimates distribution are displayed in Figure 7. The pyramidal age structures differ radically from the much older distribution of the non-Indigenous population. The age structure of 1971, shaped by high fertility and mortality, has gradually become less youth-dominated as fertility and mortality have fallen. Even in 2011, however, it remained very youthful relative to the non-Indigenous population with a median age of 23 and only 3% of the population aged 65 years and over. The estimates clearly demonstrate a population a long way off completing its demographic transition to low fertility and mortality. Population estimates by age and sex for every fifth year between 1966 and 2011 form part of the data deposit with the Australia Data Archive.

A comparison of the new estimates by age with those published elsewhere reveals some interesting differences and similarities. They are very similar to the Condon et al. [18] estimates, except at the very highest ages in 1991, 1996 and 2001, which is probably due to slight over-estimation in the 2001 ERPs which formed the starting point for Condon et al. The new estimates are marginally higher for most age groups than the ERPs, and, not surprisingly, considerably higher than the census counts at every age group. However, the proportional age distributions of census counts are quite close to the new estimates from 1976 onwards, especially when the problematic 0–4 age group is excluded. Figure S1 shows these similarities.

The new dataset probably represents the most reliable population data produced for the Northern Territory Indigenous population to date. In addition to the conceptual strength of consistent population accounts described earlier, the data generally exhibit expected patterns and relationships. For example, the overall shape of the population age structures look reasonable, and the age profiles of mortality rates are also free of obvious flaws (such as implausible changes in trend at the highest ages). Nevertheless it is cautioned that the new numbers remain *estimates* and almost certainly contain some errors derived from imperfect 2011 populations, deaths and net migration inputs, and the assumption of zero ethnic mobility. One potential problem is revealed by the high sex ratios in some older ages in the earlier years of the dataset (e.g. age group 70–74 in 1966). Although there appear to be cohort effects because the high ratios shift to older ages over time, they may still be the result of data deficiencies.

### Life expectancy and mortality

**Figure 8:**
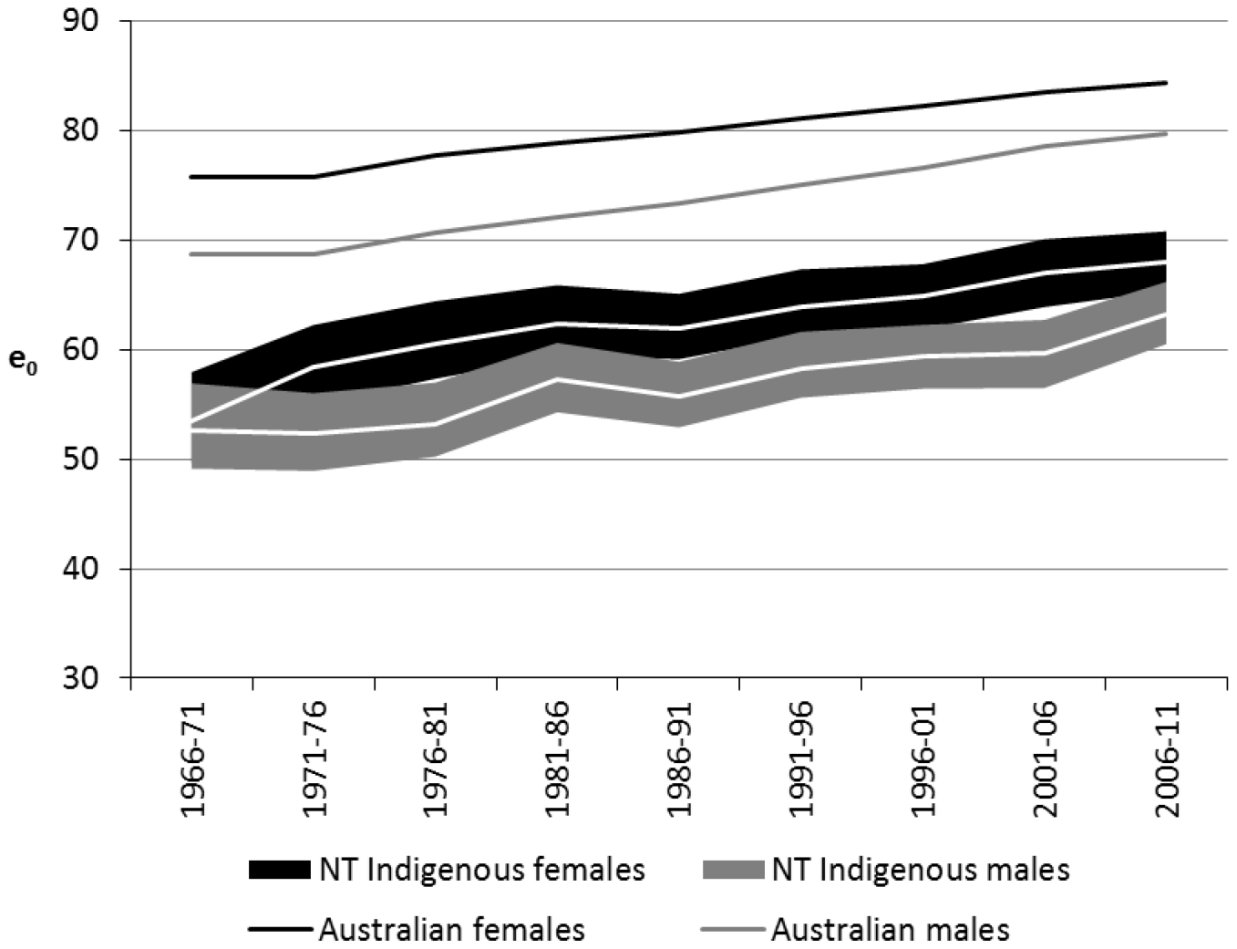
Estimates of Northern Territory Indigenous life expectancy at birth, 1966–2011.

Life expectancy at birth for the Northern Territory Indigenous population is plotted in Figure 8, together with equivalent trends for the Australian population as a whole. Abridged Indigenous life tables from this study form part of the data deposit with the Australian Data Archive. The figure shows life expectancy at about 53 years for both Indigenous males and females in the late 1960s, increasing to 68 years for females and 63 years for males by 2006–2011, with 95% confidence intervals spanning about 8 years at the start of the study period and a little over 5 years most recently. From a positive perspective, these trends represent a significant improvement over four decades and demonstrate the effectiveness of efforts to combat poor health and high mortality.

However, there is no disguising how low these figures are; the 2006–2011 Indigenous life expectancies were experienced by the Australian population as a whole in the early 1930s [37]. Moreover, the difference with all-Australian life expectancy has shown little sign of narrowing over time. Over the last 40 years the life expectancy gap for both males and females has fluctuated around 17 years. Only between 1966–71 and 1971–76 was there some narrowing of the gap for females only (Figure 8). The proximate determinants of the gap are revealed by statistics on mortality by cause of death, which for nearly all the major causes is much higher for Indigenous people [38]. The underlying influences are harder to determine but are believed to relate to disadvantage in education, household income, unhealthy behaviours, emotional wellbeing, community cohesion, and racial discrimination [39]. On the basis of the trends shown in Figure 8 the prospects for closing the gap in life expectancy within a generation – at least for the Northern Territory Indigenous population – do not look encouraging.

The adjustment of the 2011 0–4 year old population has had negligible impact on the life tables. Had the original 0–4 ERP been used in the life table calculations then 2006–11 life expectancy at birth would have differed by less than 0.1 years for both females and males.

How do these life expectancy at birth figures compare with those published elsewhere? Life tables calculated for an earlier paper [40] using the Condon et al. [18] deaths and population estimates put life expectancy for 1996–2001 at 66.0 and 59.8 years for females and males respectively, whilst the new figures for that period are slightly lower at 64.9 and 59.4 years. The differences are primarily due to lower mortality rates in the elderly ages in the earlier life tables, the result of larger populations above age 75 in the 2001 Indigenous ERPs which formed the starting point of the Condon et al. estimates. ABS Indigenous life tables for the Northern Territory put life expectancy at birth at 69.4 and 61.5 years for females and males respectively in 2005–07, and 68.7 and 63.4 years for 2010–12 [15]. Taking an average of these two sets of figures gives 69.1 and 62.5 years for the 2006–11 intercensal period, which compare with 68.0 and 63.2 years calculated for this paper.

In contrast to life expectancy at birth, under-five mortality has improved dramatically. Female and male rates have traced similar paths, both declining from around 25 per 1000 in 1966–1971 to 3 per 1000 by 2006–2011 (illustrated in Figure S2). Over these decades the gap with all-Australian under-five mortality has narrowed appreciably, down from 7.8 to 3.0 times the all-Australian rate for females, and from 5.6 to 2.6 times for males. Mortality rates have also fallen for all other age groups for females and nearly all other ages for males. However, at almost every age other than 0–4 years the gaps with all-Australian mortality rates have widened. A major public health challenge remains.

## Conclusions

This paper has presented a new set of population and life expectancy estimates for the Indigenous population of Australia's Northern Territory. Spanning a 45 year period to 2011, they represent the longest internally consistent Indigenous demographic time series in Australia. They were generated by a combination of methods in an attempt to obtain the most plausible and consistent dataset possible given the limitations of the original data. Population estimates were created by applying both reverse and forward survival in order to achieve realistic sex ratios and consistency between births and populations aged 0–4 and 5–9. Adjustments for the latter were based on new census-based evidence on reported Indigenous status changes occurring in the early childhood ages. An important feature of both the Indigenous population estimates and life expectancies is the incorporation of confidence intervals to emphasise to users the uncertainty of the statistics.

The new datasets should be useful in a number of ways. First, they provide a set of denominators for a wide range of demographic measures, such as life expectancy at birth and under-five mortality, as well as other socio-economic indicators. Importantly, mortality rates maintain numerator-denominator consistency. The paper showed that whilst under-five mortality has fallen markedly, life expectancy at birth has only risen in line with all-Australian life expectancy with no signs of the gap narrowing. Importantly, the gap in mortality rates at age groups above ages 0–4 has increased. Second, the new estimates facilitate a better understanding of long-run demographic change occurring in the Northern Territory Indigenous population. They enable crude rates of growth, birth, death and net interstate migration to be calculated, age structure change to be examined, and population accounts free of any error of closure to be created, and comparisons of all these with the population of Australia as a whole, the non-Indigenous population, as well as cross-national comparisons with Indigenous populations in other countries. Third, the new data offer a robust set of foundations on which to prepare population projections. In addition, the reported Indigenous status changes between births, 0–4 and 5–9 year olds described in the paper serve as a useful reminder that projections should also include a mechanism to handle these changes.

In conclusion, Indigenous demographic statistics in Australia have gradually improved over many decades, but much remains to be done to close the quality and coverage gap with the population as a whole. The Northern Territory's Indigenous demographic data are superior to those for the other states and territories of Australia, yet still suffer from quite a considerable degree of uncertainty. It would be good to see future developments in Indigenous population statistics involving more data linkage between census, registration and administrative datasets, thereby reducing uncertainty. In the meantime it is hoped the population and life expectancy estimates presented in this paper will be of use to researchers and policymakers.

## Supporting Information

Figure S1 **Age distribution of the Northern Territory Indigenous population at ages 5+ as revealed by the new estimates and census counts.**
(TIF)

Figure S2 **Under-five mortality rates, Northern Territory (NT) Indigenous population, 1966–2011.**
(TIF)

Table S1 **Sensitivity to variations in relative standard deviation assumptions for random error in death and net migration sample paths.**
(DOC)
