# Supplementary figures and images for: New Population and Life Expectancy Estimates for the Indigenous Population of Australia's Northern Territory, 1966–2011
Source: PLoS One. 2014 May 27;9(5):e97576. doi: 10.1371/journal.pone.0097576 (PMC4035253; doi:10.1371/journal.pone.0097576)

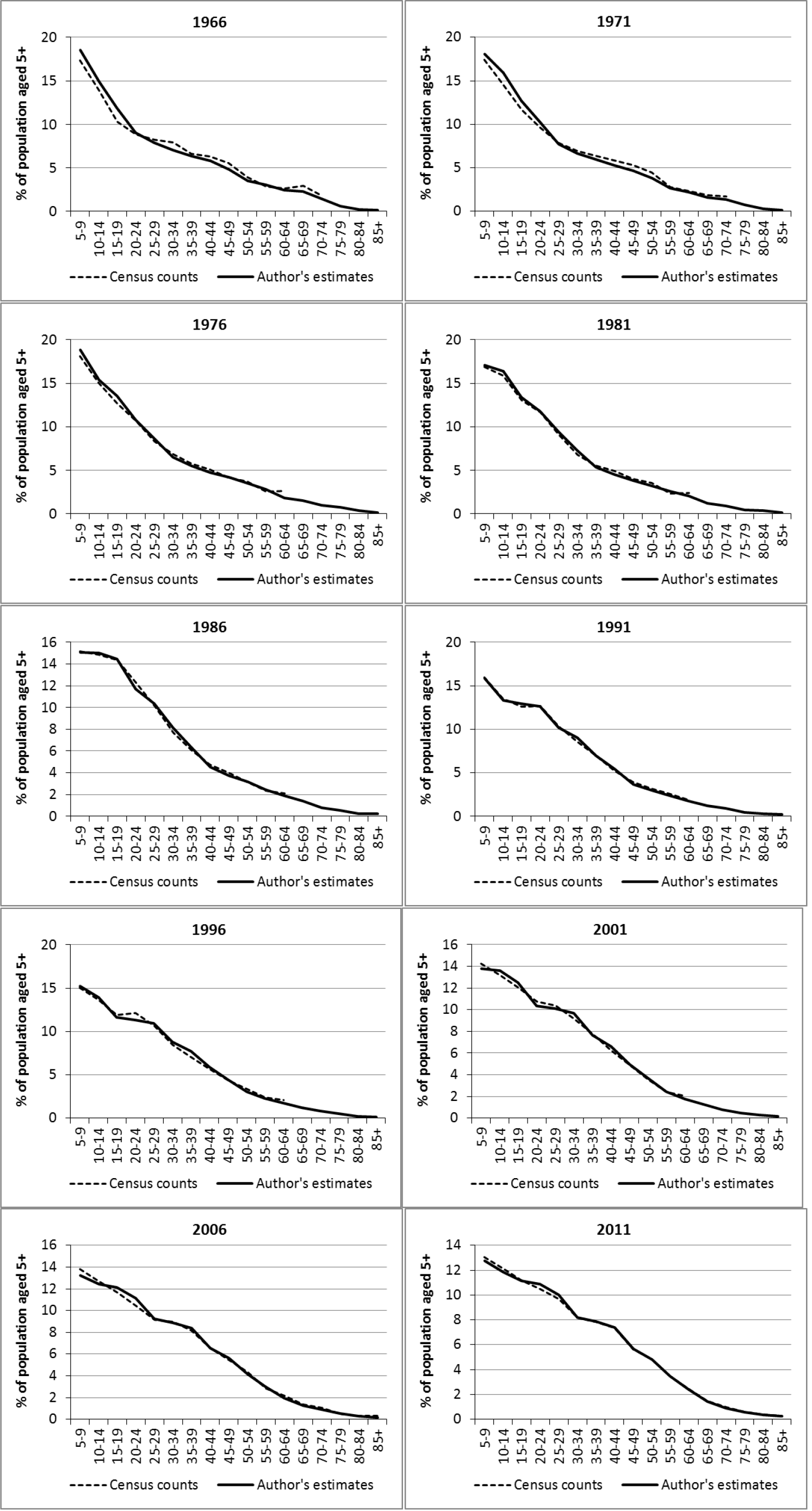

Supplement: Figure S1 — Age distribution of the Northern Territory Indigenous population at ages 5+ as revealed by the new estimates and census counts. (TIF) [file pone.0097576.s001.tif]

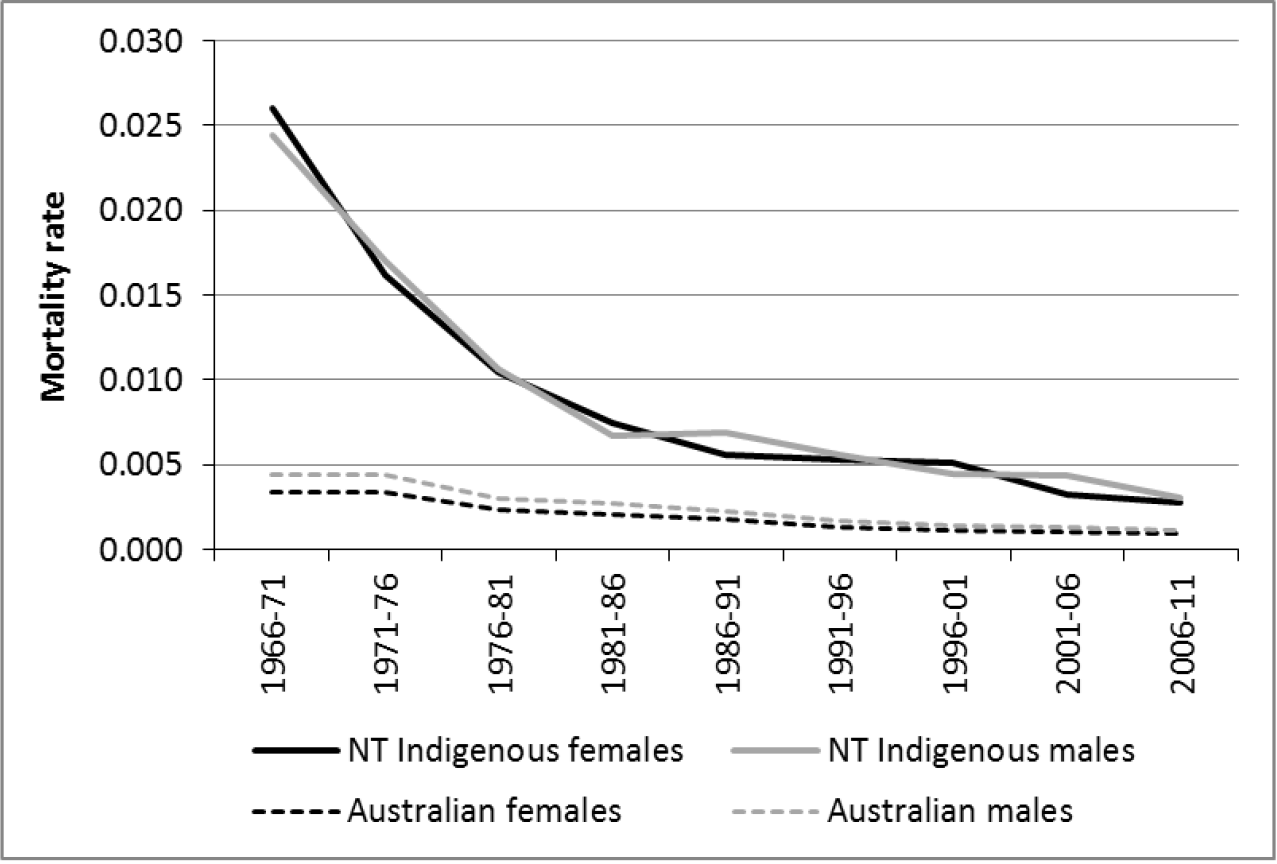

Supplement: Figure S2 — Under-five mortality rates, Northern Territory (NT) Indigenous population, 1966–2011. (TIF) [file pone.0097576.s002.tif]
