# Supplementary material for: New Population and Life Expectancy Estimates for the Indigenous Population of Australia's Northern Territory, 1966–2011
Source: PLoS One. 2014 May 27;9(5):e97576. doi: 10.1371/journal.pone.0097576 (PMC4035253; doi:10.1371/journal.pone.0097576)
Supplement: Table S1 — Sensitivity to variations in relative standard deviation assumptions for random error in death and net migration sample paths. (DOC) [file pone.0097576.s003.doc]

**Supporting Information**

**Table S1**: Sensitivity to variations in relative standard deviation assumptions for random error in death and net migration sample paths

|  | Deaths and net migration relative standard deviations | | |
| --- | --- | --- | --- |
|  | Chosen:  Deaths: 0.02  Net migration: 0.10 | Halved:  Deaths: 0.01  Net migration: 0.05 | Doubled:  Deaths: 0.04  Net migration: 0.20 |
| *Width of total population 95% confidence interval (persons)* | | | |
| 1966 | 2,336 | 1,620 | 4,210 |
| 1991 | 4,062 | 4,020 | 4,403 |
| 2011 | 7,059 | 7,066 | 7,080 |
| *Width of female^§^ life expectancy at birth 95% confidence interval (years)* | | | |
| 1966-71 | 8.3 | 6.1 | 14.9 |
| 1986-91 | 6.0 | 4.6 | 10.2 |
| 2006-11 | 5.4 | 4.7 | 7.0 |

Note: Because confidence intervals were generated by 5,000 simulations, slightly different results are created with different runs of the model.

§ Male life expectancy at birth confidence intervals are of similar width so are not shown.
